# Prevalence and management of sleep disturbance in adults with primary brain tumours and their caregivers: a systematic review

**DOI:** 10.1007/s11060-023-04270-1

**Published:** 2023-03-02

**Authors:** Jason A. Martin, Nicolas H. Hart, Natalie Bradford, Fiona Naumann, Mark B. Pinkham, Elizabeth P. Pinkham, Justin J. Holland

**Affiliations:** 1 Faculty of Health, School of Exercise and Nutrition Science, Queensland University of Technology, Brisbane, Australia; 2 Faculty of Health, School of Sport, Exercise and Rehabilitation, University of Technology Sydney (UTS), Sydney, Australia; 3 Faculty of Health, Cancer and Palliative Care Outcomes Centre, Queensland University of Technology, Brisbane, Australia; 4 School of Medical and Health Sciences, Exercise Medicine Research Institute, Edith Cowan University, Perth, Australia; 5 College of Nursing and Health Sciences, Caring Futures Institute, Flinders University, Adelaide, Australia; 6 Institute for Health Research, The University of Notre Dame Australia, Perth, Australia; 7 Faculty of Health, Southern Cross University, Gold Coast, Australia; 8 Radiation Oncology, Division of Cancer Services, Princess Alexandra Hospital, Brisbane, Australia; 9 Physiotherapy, Clinical Support Services, Princess Alexandra Hospital, Brisbane, Australia; 10 Faculty of Health, School of Nursing, Queensland University of Technology, Brisbane, Australia; 11 Faculty of Medicine, University of Queensland, Brisbane, Australia

**Keywords:** Sleep disturbance, Primary brain tumour, Caregivers, Neuro-oncology, Quality of life, Exercise

## Abstract

**Purpose:**

The aims of this systematic review were to (1) examine the prevalence, severity, manifestations, and clinical associations/risk factors of sleep disturbance in primary brain tumour (PBT) survivors and their caregivers; and (2) determine whether there are any sleep-focused interventons reported in the literature pertaining to people affected by PBT.

**Methods:**

This systematic review was registered with the international register for systematic reviews (PROSPERO: CRD42022299332). PubMed, EMBASE, Scopus, PsychINFO, and CINAHL were electronically searched for relevant articles reporting sleep disturbance and/or interventions for managing sleep disturbance published between September 2015 and May 2022. The search strategy included terms focusing on sleep disturbance, primary brain tumours, caregivers of PBT survivors, and interventions. Two reviewers conducted the quality appraisal (JBI Critical Appraisal Tools) independently, with results compared upon completion.

**Results:**

34 manuscripts were eligible for inclusion. Sleep disturbance was highly prevalent in PBT survivors with associations between sleep disturbance and some treatments (e.g., surgical resection, radiotherapy, corticosteroid use), as well as other prevalent symptoms (e.g., fatigue, drowsiness, stress, pain). While the current review was unable to find any sleep-targeted interventions, preliminary evidence suggests physical activity may elicit beneficial change on subjectively reported sleep disturbance in PBT survivors. Only one manuscript that discussed caregivers sleep disturbance was identified.

**Conclusions:**

Sleep disturbance is a prevalent symptom experienced by PBT survivors, yet there is a distinct lack of sleep-focused interventions in this population. This includes a need for future research to include caregivers, with only one study identified. Future research exploring interventions directly focused on the management of sleep disturbance in the context of PBT is warranted.

**Supplementary Information:**

The online version contains supplementary material available at 10.1007/s11060-023-04270-1.

## Introduction

Persistent sleep dysfunction negatively impacts quality of life (QoL) in people affected by cancer, with evidence suggesting an association between sleep quality and other health-related and cancer-specific outcomes, including survival [1, 2]. While previous reviews have reported sleep outcomes in the general cancer populations, people with primary brain tumours (PBT) are rarely included in data synthesis despite the symptom being increasingly recognized in clinical care; this is likely due to the rarity of PBT, representing ~ 1.5% of all cancers diagnosed [1, 3, 4]. Beyond the burden placed on PBT survivors, the physical and emotional burden of becoming a family caregiver (herein referred to as “caregivers”) for a person with PBT may also lead to sleep disturbance. The survivor-caregiver dyad is a critical relationship, with caregivers heavily involved in care coordination and support. However, this role directly exposes caregivers to numerous factors that may directly impact sleep including interruptions to routines and schedules (e.g., attending to overnight care needs, prioritising medical assistance) and increased stress, worry and hypervigilance manifesting into psychological issues (e.g., depression, anxiety) [5, 6].

Two recent review articles [6, 7] highlight sleep disturbance as a highly prevalent and severe symptom impacting health-related quality of life (HRQoL) in PBT survivors, citing sleep disturbance as one of the most frequent and impactful symptoms reported throughout the disease trajectory. Additionally, the poor prognosis many people with PBT impacts the ability to perform detailed longitudinal studies [8, 9]. Unfortunately, there is a notable lack of understanding concerning the patterns, prevalence, severity, and risk factors for sleep disturbance in PBT survivors and their caregivers. Both reviews [6, 7] highlight major drawbacks in prior literature: (1) most evidence stems from HRQoL measures that only include a single item or question pertaining to an individual’s sleep, (2) study samples are typically small groups of people with low-grade or benign tumours following surgical resection, limiting the ability to infer results for higher grade disease, and (3) there is a distinct lack of inclusion of caregivers in prior literature. The review by Jeon et al. [6] also included studies of people with secondary (metastatic) brain cancer, further limiting the inferences that can be made from the general results of this review for PBT survivors specifically.

Our systematic review assimilates the latest evidence to understand the prevalence and risk factors of sleep disturbance in people affected by PBT. Jeon et al. [6] reported findings between January 1990 and September 2015. Since then, attention towards neuro-oncology HRQoL has resulted in augmented clinical and supportive care measures, justifying an updated review. Our primary aim was to examine the prevalence, severity, and manifestations of sleep disturbance in PBT survivors and their caregivers and explore potential risk factors and clinical determinants of sleep disturbance in these two groups. A secondary aim was to determine whether there are any sleep-focused interventions reported in the literature pertaining to people affected by PBT.

## Methods

### Search strategy and eligibility criteria

We conducted a systematic review in accordance with the Preferred Reporting Items for Systematic Reviews and Meta-Analysis (PRISMA) 2020 guidelines [10], and registered with the international register for systematic reviews (PROSPERO: CRD42022299332). Searches were conducted using the PubMed, EMBASE, Scopus, PsychINFO, and CINAHL electronic databases. The search strategy included keywords or medical sub-headings related to sleep and primary brain tumours, appropriately adapted for each database. Database limiters were applied, where applicable, to identify publications in English with adult subjects between September 2015 and May 2022 to avoid cross-over with the work by Jeon et al. [6] and limited only to PBT survivors and their caregivers. Additional manual searches for relevant manuscripts not identified in the initial search strategy and/or previously included in earlier reviews were performed on reference lists of eligible manuscripts; these additional manual searches were not limited by date range though were subject to the other database limiters. To improve the translation of the search strategy across multiple databases, the Bond Universities Systematic Review (SR) Accelerator [11] Polyglot Search Translator [12] was used. Studies reporting information regarding sleep disturbance or interventions to assist with improving or managing sleep disturbance in adults (≥ 18 years) diagnosed with PBT during or post-treatment, and their caregivers were included. Tools we considered for reporting sleep disturbance included:

1. Validated self-report sleep assessments;
2. Sleep recording device that provides objective sleep data;
3. Sleep diary or log with ≥ 7 sleep–wake cycles;
4. A validated HRQoL study reporting ≥ 1 sleep item;
5. A validated psychological assessment reporting ≥ 1 sleep item;
6. A validated symptom assessment reporting ≥ 1 sleep item.

The results from all database searches were imported to a combined library in EndNote (X9). The library was then imported to the SR Accelerator [11], where duplicates were removed using the Deduplicator tool. One author (JM) screened all titles and abstracts based on the above criteria using the SR-Accelerators Screenatron tool [11]. Full-text articles were then assessed by two independent reviewers (JM, AD); any discrepancies were discussed and resolved by a third author (JH).

### Quality appraisal

Articles eligible for inclusion were assessed using the Joanna Briggs Institute (JBI) critical appraisal tool [13] relevant for each study design. The JBI critical appraisal tool is a standardised methodology used to assess the quality of studies for risk of bias and overall quality of individual studies based on a number of criteria including study design, sample size, and method of data collection. Quality appraisal was conducted independently by JM and AD with arbitration by JH when required. Studies were not excluded based on their quality appraisal.

### Data extraction and statistical analysis

Relevant information from eligible studies was recorded in a data extraction spreadsheet, including publication information (title, authors, publication year), study design, sample size, demographic information (e.g., age, sex, cancer type, treatment status), and study aims and outcomes (e.g., sleep measures reported, sleep outcomes). Due to the small and heterogenous sample sizes recruited for included studies and varying methodologies, a meta-analysis was not performed. Studies were categorised as intervention studies, descriptive sleep studies, or HRQoL studies according to the primary focus or objective of each study. Sleep related outcomes were extracted as reported in the original manuscripts; for intervention studies, a *p* value of ≤ 0.05 was considered statistically significant unless stated otherwise.

## Results

### Inclusion of studies

**Fig. 1:**
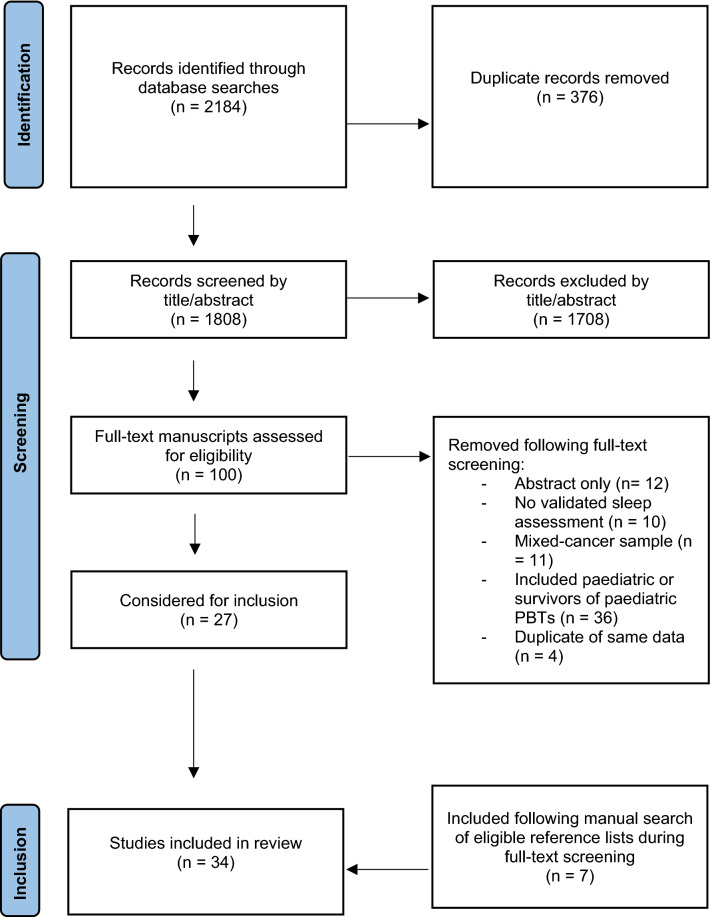
PRISMA flow chart

A total of 2184 manuscripts were evaluated for title and abstract screening, which was reduced to 100 articles for full text screening following removal of duplicates. Following full-text review, 27 articles of varying quality were included in the systematic review. Following further manual searches, an additional 7 articles were included. Therefore a total of 34 manuscripts were included in this review (Fig. 1).

### Characteristics of included studies

All 34 included articles were published between 1998 and 2022, consisting of 16 cross-sectional studies [14–29], 7 randomised control trials (RCT) [30–36], 4 quasi-experimental studies [37–40], and 7 cohort studies [41–47]. The results of the included articles were informed by 4,694 PBT survivors and 120 caregivers, with sample sizes ranging from 12 to 621 with an age range of 18–86 years. Most articles reported gender distribution, noting PBT survivors were predominantly male (52%) and caregivers female (71%). Ten studies exclusively included high-grade glioma (HGG) samples [17, 24, 25, 30, 36, 37, 40, 43, 46, 47], while seven exclusively included low-grade glioma (LGG) samples [21, 23, 27, 31, 35, 39, 42]. Seventeen studies involved mixed samples of LGG and HGG [14–16, 19, 20, 22, 26, 28, 29, 32–34, 38, 39, 41, 44, 45].

Sleep was primarily assessed using validated subjective questionnaires, including the Pittsburgh Sleep Quality Index (PSQI) [19, 21, 29, 33, 37], the Insomnia Severity Index (ISI) [29, 45], the Epworth Sleepiness Scale (ESS) [21], the Athens Insomnia Scale (AIS) [21], the General Sleep Disturbance Scale (GSDS) [17], and the Brief Sleep Disturbance Scale (BSDS) [32]. Furthermore, validated symptom or outcome measures reporting ≥ 1 sleep item included the NCCN Distress thermometer [20], the PROMIS Clinical Outcomes Assessment (V1) [22], the Fact-BR [36], and the Common Terminology Criteria for Adverse Events (CTCAE) [26]. In addition to these validated sleep or symptom assessment tools, validated HRQoL assessments that included at least 1 sleep-related item were also reported, including the EORTC QLQ-C30 [16, 23–25, 27, 28, 30, 31, 34, 35, 39–47] & BN20 [16, 18, 24, 25, 28, 30, 31, 39, 45, 47] modules, describing insomnia and drowsiness, respectively, as well as the MDASI-BT [14, 15, 39]. Finally, a small number of studies also included objective assessments including actigraphy [21] and polysomnography (PSG) [38].

Articles were categorised into three groups based on the following criteria: (1) reported validated sleep tool or a validated symptom or outcome assessment that reported ≥ 1 sleep measure (Table 1); (2) reported a HRQoL assessment (Table 2); or (3) included intervention studies that reported a validated sleep measure, HRQoL measure, or a validated symptom or outcome assessment that reported ≥ 1 sleep measure (Table 3). In total, 9 (25%) included articles had a primary focus on the prevalence, severity or presentation of sleep disturbance in PBT survivors [19–22, 26, 29, 30, 38], with the remaining articles having a primary focus on HRQoL [14–18, 23–25, 28, 30–37, 39–48].

**Table 1:** Studies reporting a validated sleep measure and/or a validated symptom/outcome assessment that reported ≥ 1 sleep measure

Study	Sample	Purpose	Sleep assessments	Sleep assessment result	Conclusion
Fox et al. (2007) [18]	n = 73 (39 M, 34 F) Adult survivors of HGGs, mean age 46 ± 13.03 years Avg time since diagnosis = 46 months 45.2% GBM 26% anaplastic astrocytoma 20.5% WHO III oligoastrocytoma 90% undergone surgery, 47% undergone chemoradiotherapy	To describe co-occurring symptoms, quality of life and functional status in patients with HGGs	GSDS	Mean ± SD Sleep Disturbance = 48.02 ± 17.86	100% of participants in the experienced sleep disturbance, occurring in symptom clusters with depression, fatigue, cognitive impairment, pain, functional status, and quality of life
Huang et al. (2020) [20]	n = 358 Post-operative glioma patients, mean age 44.64 ± 11.07 Mean post-op time of 4.41 ± 2.57 years	To investigate sleep quality in postoperative glioma patients	PSQI	*Overall PSQI results* Mean ± SD total PSQI Score = 5.19 ± 3.39 PSQI > 5 = 37% PSQI > 7 = 37.7% Poor to Quite Poor Sleep Quality = 21% > 30 min to fall asleep = 29.2% > 60 min to fall asleep = 10% *Post-op Mean* ± *SD PSQI Scores* < 2years post-op = 5.56 ± 2.39 2-5years post op = 4.53 ± 2.30 > 5years post-op = 3.95 ± 1.78	Post-operative glioma patients have a high prevalence of sleep disturbance
Kopelke et al. (2022) [21]	n = 57 (32 M, 25 F) WHO II-IV glioma patients scheduled for radiochemotherapy, median age 59 years	Determine the prevalence of sleep problems in glioma patients and identify associated risk factors	NCCN Distress Thermometer	Prevalence of sleep disturbance = 66.7%	Retrospective analysis found that 19 of 57 patients stated sleep problems prior to their planned course of radiochemotherapy
Langegard et al. (2021) [46]	n = 266 (86 M, 73 F) Adult PBT patients divided into malignant or benign tumour groups: 159 malignant tumours, mean age 44 ± 12.4 years 107 benign tumours, mean age 56 ± 13.4 years	Describe and compare HRQOL, including acute symptom experiences and associated factors, in patients with malignant and benign brain tumours treated with PBT	ISI	*Mean* ± *SD ISI Results for each time point* *Baseline* Malignant 4.59 ± 6.30 Benign = 5.75 ± 7.49 *Middle of treatment* Malignant = 5.04 ± 6.78 Benign = 5.56 ± 7.39 *End of treatment* Malignant = 5.37 ± 7.11 Benign = 6.42 ± 8.59 *1-month post-treatment* Malignant = 5.07 ± 6.90 Benign = 5.78 ± 7.92 *3-months post-treatment* Malignant = 3.86 ± 6.31 Benign = 4.54 ± 7.12	While deteriorations in HRQOL were noted between baseline (during treatment) and 3-months post-treatment, there were improvements noted in insomnia in both study groups; in the benign tumour group, significant improvement in insomnia was noted. However, both groups experienced significant deteriorations in fatigue
Lin et al. (2021) [22]	n = 77 (22 M, 55 F) Patients recently diagnosed with pituitary tumour (n = 33) or WHO I meningiomas (n = 44), mean age 49.16 years	To explore the prevalence of sleep disturbance and its effects on quality of life in adults with pituitary tumour or meningioma	CAIS CESS CPSQI Actigraph – Mini Motionlogger (at least 3 days); sleep log co-ordinated with Actigraph	*Questionnaire Mean* ± *SD Scores* CPSQI = 11.53 ± 5.38 CAIS = 8.00 ± 4.48 CESS = 2.69 ± 3.66 Prevalence was not significantly different between groups (p > 0.05) *Actigraphy Mean* ± *SD sleep scores* TST = 387.2 ± 124.7 min SOL = 4.1 ± 2.42 min WASO = 32.5 ± 16.56 min SE = 92.9 ± 3.25% ~ 60% of participants experienced slight circadian rhythm disruption	Mean CPSQI, CAIS and CESS scores reflect that participants experienced poor sleep quality (81.8% of participants) and insomnia (46.8% of participants), but not excessive daytime sleepiness (6.5% of all participants). The differences in prevalence between the two groups was not significant (p > 0.05). There were no significant between-group differences in measures of TST (p = 0.74), SOL (p = 0.85) or SE (p = 0.98) Acceptable score ranges compared to normative data were recorded via actigraphy
Miklja et al. (2021) [23]	n = 38 (13 M, 15 F) Adult high- and low-grade glioma patients; split into high (n = 27) or low tolerance (n = 11) to exercise Avg age = 50 years Avg age High tolerance = 49 years; Avg age Low tolerance = 53.6 years Glioma Grade: High Grade – 20 high tolerance (52.6%), 8 low tolerance (21.1%) Low Grade – 7 high tolerance (18.4%), 3 low tolerance (7.9%)	To determine if tolerance and experience of exercise impacts HRQOL in adult high- and low-grade glioma patients, particularly sleep and fatigue	PROMIS Version 1.0	*Sleep t score* Low Tolerance = 56.4; High Tolerance = 48.7; p = 0.02	Glioma patients with low tolerance to exercise had more sleep disturbance than glioma patients with higher tolerance to exercise
Panciroli et al. (2022) [39]	n = 12 (10 M, 2 F) PBT patients undergoing radiotherapy, mean age 48.5 years n = 8 healthy controls, mean age 38.5 years	Assess if the radiotherapy dose decreases salivary melatonin levels as well as the quality of life and sleep in brain tumour patients	PSG	*Overall Average PSG Results* *Baseline* TST: 319 min SE: 85.1% WASO = 39.2 min SL = 8.8 min *Follow-up (End of Radiotherapy)* TST: 308 min SE: 81.3% WASO = 35.8 min SL = 14.8 min *Last (2-months post radiotherapy)* TST: 314.5 min SE: 83.3% WASO = 33.5 min SL = 9 min *Overall Average Melatonin Results (pmol/L)* *Baseline* Night = 19.8 Morning = 35.2 *Follow-up* Night = 22.1 Morning = 30.8 *Last* Night = 16.7 Morning = 32.7	No statistically significant differences in melatonin or PSG outcomes were found according to radiotherapy dose delivered in the present study. There were no significant differences observed between patient baseline PSG results and healthy controls
Robertson et al. (2016) [27]	n = 340 (119 M, 62 F) Recurrent glioma patients, mean age 48.4 ± 11.7 years, majority diagnosed with HGG (87.1%)	To better understand insomnia in recurrent glioma patients	CTCAE	Insomnia documented in 46.8% (n = 159) of patients	Retrospective analysis showed insomnia is a common complaint in recurrent glioma patients irrespective of grade
Willis et al. (2022) [30]	n = 119 (60 M, 58 F, 1 unspecified) Mixed PBT patients, mean age 52.60 ± 15.39 years Meningioma – 18 (15.1%) Astrocytoma – 27 (22.7%) GBM – 44 (37%) Other/unknown tumour type – 9 (7.6%)	Determine the prevalence and associated risk factors of sleep disturbance among PBT patients	PSQI ISI	*Mean* ± *SD PSQI Results* Overall PSQI = 8.81 ± 6.70 Poor sleep quality (PSQI > 5) = 61.5% Poor SE (< 85%) = 52.1% *Mean* ± *SD ISI Results* Overall ISI = 7.19 ± 4.27 No clinically significant insomnia = 44.6% Sub-clinical insomnia = 31.4% Moderate insomnia = 17.4% Severe insomnia = 4.1%	All participants with insomnia as measured by the ISI endorsed poor sleep quality as measured by the PSQI

*CAIS* Chinese Athens Insomnia Scale; *CESS* Chinese Epworth Sleepiness Scale; *CPSQI* Chinese Pittsburgh Sleep Quality Index; *CTCAE* Common Terminology for Adverse Events; *GBM* Glioblastoma Multiforme; *GSDS* General Sleep Disturbance Scale; *HGG* High-Grade Glioma; *HRQOL* Health related quality of life; *ISI* Insomnia Severity Index; *NCCN* National Comprehensive Cancer Network; *PBT* Primary brain tumour; *PSG* Polysomnography; *PSQI* Pittsburgh Sleep Quality Index; *SE* Sleep Efficiency; *SOL* Sleep Onset Latency; *TST* Total Sleep Time; *WASO* Wake After Sleep Onset; *WHO* World Health Organisation

**Table 2:** Insomnia and drowsiness subscale scores for HRQOL assessments

Study	Sample	Purpose	HRQOL Tool	Insomnia^a^/disturbed sleep^c^ Mean ± SD	Drowsiness^b,c^ Mean ± SD
Ahn et al. (2021) [31]	n = 84 (46 M, 38 F) WHO III Glioma patients Mean age 44.7 ± 13.2 years	Investigate the effect of concurrent and adjuvant temozolomide on HRQOL Patients assigned to concurrent chemoradiotherapy with temozolomide followed by 6 cycles of adjuvant temozolomide (arm A; n = 40) and radiotherapy alone (arm B; n = 44)	EORTC QLQ-C30 & BN20	*	*
Armstrong et al. (2012) [16]	n = 115 dyads (Patients – 73 M, 42 F; Caregivers – 31 M, 84 F) Mixed PBT patient/caregiver dyads Mean caregiver age = 49.8, Mean patient age = 48.2 years	Assess the congruence of symptom reporting in PBT patient/caregiver dyads	MDASI-BT	Patient score = 2.35 ± 2.89 Caregiver score = 3.03 ± 3.18	Patient score = 2.77 ± 2.76 Caregiver score = 3.03 ± 2.82
Armstrong et al. (2016) [15]	n = 621 (366 M, 251 F) Mixed PBT patients Median age 47 years (range = 18–84)	Describe and compare symptoms identified by the CMTP and SOAPP study in PBT patients	MDASI-BT	22% reported	28% reported
Bunevicius et al. (2013) [17]	n = 100 (29 M, 71 F) PBT patients admitted for elective brain surgery, mean age = 58 ± 14 years Meningioma = 46 HGG = 19 LGG = 2 Pituitary tumour = 16 Acoustic neuroma = 7 Other = 10	Evaluate the psychometric properties of the EORTC QLQ-BN20 brain tumour specific module in Lithuanian brain tumour patients	EORTC QLQ-C30 & BN20	44 ± 40.4	23.3 ± 21.2
Bunevicius et al. (2017) [42]	n = 63 (21 M, 42 F) PBT patients prior to surgery, mean age = 55.5 ± 13.8 years HGG = 16 LGG = 3 Meningioma = 25 Pituitary adenoma = 5 Acoustic neuroma = 8 Other = 9	Investigate the association of normal and abnormal thyroid hormone concentrations with HRQOL of patients with primary brain tumours	EORTC QLQ-C30	*	–
Dirven et al. 2018 [32]	n = 195 (110 M, 85 F) LGG patients, primarily ≥ 40 years of age (63.6%) Tumour location: Front = 77 Temporal = 38 Parietal = 16 Occipital = 2 Multifocal = 45 Other = 16 Missing = 1	Investigate whether the size of the target volume of RT is independently associated with HRQOL in LGG patients	EORTC QLQ-C30 & BN20	21.4 ± 28.4	24.7 ± 26.3
Fedorko et al. (2018) [43]	n = 32 (10 M, 22 F) Patients following surgical treatment of lesions with the pineal region, mean age = 39 years (range = 18–73 years)	Assess the therapeutic effect of surgical resection of pineal region lesions	EORTC QLQ-C30	Pre-op = 53.33 ± 43.80 Post-op = 33.33 ± 34.20	–
Habets et al. (2014) [44]	n = 32 (19 M, 13 F) Long-term anaplastic oligodendroglioma and oligoastrocytoma with 1p/19q codeletion and non-1p/19q deletion survivors, mean age = 56.7 ± 8.3 years Anaplastic oligodendroglioma = 22 Anaplastic oligoastrocytoma = 10	Evaluate HRQOL outcomes and cognitive functioning in long-term survivors of anaplastic oligodendroglioma and oligoastrocytoma with 1p/19q codeletion and non-1p/19q deletion tumours	EORTC QLQ-C30	*	–
Hansen et al. (2021) [19]	n = 81 (54 M, 27F) Glioma patients R Side mean age = 56.8 ± 13.1 years L side mean age = 55.3 ± 13.3 years WHO II = 11 WHO III = 10 WHO IV = 60	Comparison of early disease symptomology, functional performance and HRQOL outcomes based on hemispheric location (L vs R) in adult glioma patients	EORTC QLQ-C30 & BN20	Right side = 20.0 ± 27.0 Left side = 29.6 ± 30.6	Right side = 41.5 ± 34.9 Left side = 38.0 ± 29.0
Kiebert et al. (1998) [36]	n = 379 LGG patients High dose group primarily ≥ 45 years (36%) Low dose group primarily 35–44years (34%)	Assessment of quality-of-life outcomes following a randomised phase III trial on dose response of radiation therapy comparing high-dose (59.4 Gy in 6.5 weeks) versus low-dose (45 Gy in 5 weeks) radiotherapy with conventional techniques	EORTC QLQ-C30	*	–
Langegard et al. (2019) [45]	n = 186 (88 M, 98 F) Mixed PBT patients, mean age = 48 ± 14 years	To describe the perceptions of quality of care and its association with HRQOL in PBT patients undergoing proton beam therapy	EORTC QLQ-C30	Baseline = 24.1 ± 30.0 3 weeks = 27.7 ± 30.1 6 weeks = 28.2 ± 32.1	–
Langegard et al. (2021) [46]	n = 266 (86 M, 73 F) Adult PBT patients divided into malignant or benign tumour groups: 159 malignant tumours, mean age 44 ± 12.4 years 107 benign tumours, mean age 56 ± 13.4 years	Describe and compare HRQOL, including acute symptom experiences and associated factors, in patients with malignant and benign brain tumours treated with proton beam therapy	EORTC QLQ-C30 & BN20	Baseline: Malignant = 27.0 ± 28.6 Benign = 21.5 ± 29.8 Mid: Malignant = 25.8 ± 28.5 Benign = 28.7 ± 30.9 End: Malignant = 28.9 ± 30.0 Benign = 31.2 ± 34.0 1-month post: Malignant = 26.8 ± 30.6 Benign = 24.9 ± 30.7 3-month post: Malignant = 21.6 ± 27.3 Benign = 21.5 ± 30.1	Baseline: Malignant = 31.6 ± 26.7 Benign = 32.0 ± 26.8 Mid: Malignant = 34.2 ± 27.3 Benign = 34.6 ± 27.7 End: Malignant = 40.4 ± 30.5 Benign = 43.5 ± 30.7 1-month post: Malignant = 42.1 ± 31.2 Benign = 40.8 ± 30.4 3-month post: Malignant = 41.7 ± 28.7 Benign = 41.8 ± 30.6
Minniti et al. (2009) [47]	n = 43 (21 M, 22 F) Elderly GBM patients, median age = 73 years (range = 70–79 years)	Determine optimal treatment for elderly GBM patients	EORTC QLQ-C30	15.1 ± 3.4	–
Nassiri et al. (2019) [24]	n = 291 (61 M, 230 F) Patients in follow-up from resection of WHO I intracranial meningioma, mean age = 60.08 ± 11.95 years	Identify possible actionable determinants of global HRQoL in grade 1 meningioma patients	EORTC QLQ-C30	*	–
Onken et al. (2019) [25]	n = 30 (20 M, 10 F) HGG patients undergoing TTFields treatment, mean age 50 years n = 27 (19 M, 8 F) Control group, HGG patients undergoing normal treatment, mean age 47 years	Objective study comparing PROs between patients undergoing TTFields treatment and regular treatment	EORTC QLQ-C30 & BN20	*	*
Pollom et al. (2017) [48]	n = 30 (15 M, 15 F) GBM patients, median age = 66years (range = 51–86 years)	Longitudinal assessment of HRQOL in patients with glioblastoma treated on a prospective dose escalation trial of 5-fraction stereotactic radiosurgery	EORTC QLQ-C30 & BN20	35.7 ± 35.1	21.4 ± 26
Renovanz et al. (2020) [26]	n = 309 (179 M, 130 F) HGG patients, mean age = 55 ± 14 years, with WHO IV tumours (59%) Tumour localization: Frontal = 128 Temporal = 88 Parietal = 21 Occipital = 48 Other = 20 Unknown = 4	Compare HRQOL and distress between elderly and younger patients with high-grade glioma (HGG)	EORTC QLQ-C30 & BN20	≥ 65 years = 27.0 ± 32.1 < 65 years = 36.4 ± 35.2	≥ 65 years = 27.0 ± 32.1 < 65 years = 36.4 ± 35.2
Scartoni et al. (2020) [41]	n = 26 (18 M, 8 F) Recurrent GBM patients re-irradiated with active scanning proton therapy, median age at re-irradiation = 53.4 years (Range = 30–69 years)	Determine the effect of re-irradiation with active scanning proton therapy on HRQOL	EORTC QLQ-C30	*	–
Teng et al. (2021) [28]	n = 167 LGG patients following surgical resection, median time from surgery = 38 months, patients primarily diagnosed with WHO II tumours (88.02%)	Prospective, longitudinal, cross-sectional cohort study of HRQOL in LGG patients, aiming to identify actionable determinants of HRQOL	EORTC QLQ-C30	Range: 26.19 ± 26.73 to 33.33 ± 36.15	–
Umezaki et al. (2020) [29]	n = 76 (41 M, 35 F) Glioma patients, median age = 51 years (Range = 24–83 years) Majority male (53.9%) outpatients (81.6%) during follow-up observation (60.5%)	Document the quality of life of patients with glioma and clarify the impact of symptoms	EORTC QLQ-C30 & BN20	20.2 ± 28.8	36.4 ± 33.6
Waddle et al. (2019) [40]	n = 20 (10 M, 10 F) Adult glioma patients undergoing surgical resection for pituitary tumours; mean age = 51	Better understand the impact of surgery on patients' symptom burden and quality of life in the subacute post-surgical period	EORTC QLQ-C30 & BN20 & MDASI-BT	Pre-op: 30 ± 32^a^ Post-op: 40 ± 34^a^ Pre-op: 3.1 ± 2.9^c^ Post-op: 4.8 ± 3.2^c^	Pre-op: 37 ± 29^b^ Post-op: 25 ± 24^b^ Pre-op: 3.6 ± 3.4^c^ Post-op: 3.4 ± 3.0^c^

^a^EORTC QLQ-C30

^b^EORTC QLQ-BN20

^c^MDASI-BT

*Studies used HRQOL measure and discussed sleep/drowsiness outcomes, but reported data visually (e.g., graph)

*CMTP* Centre for medical technology policy; *GBM* glioblastoma multiforme; *HGG* high-grade glioma; *HRQOL* health related quality of life; *LGG* low-grade glioma; *PBT* primary brain tumour; *RT* radiation therapy; *PRO* patient reported outcomes; *SOAPP* screener and opioid assessment of patients with pain; *WHO* World Health Organisation

**Table 3:** Potential Interventions to assist with management of sleep disturbance in PBT patients and their caregivers

Study	Sample	Purpose	Intervention	Sleep assessment	Outcome
Gehring et al. (2012) [33]	n = 24 (13 M, 11 F) PBT patients Methylphenidate: mean age = 42.5 ± 10.2 years, median time since surgery (days) = 370 (103–3334) Modafinil: mean age = 54.4 ± 7.7 years, median time since surgery (days) = 1105 (315–4413) Majority of pts diagnosed with HGG (66%) following surgical resection of tumour	Open-label, pilot study examining both the general and differential efficacy of 4 weeks of methylphenidate (MPH) and modafinil (MOD) in PBT patients	Participants randomly assigned to 1 of 3 conditions: (1) 10 mg b.i.d. of methylphenidate IR (Ritalin; IR-MPH) for 4 weeks (2) 18 mg q.d. (AM) of methylphenidate SR (Concerta;SR-MPH) for 4 weeks (3) 200 mg q.d. (AM) of modafinil (Provigil; MOD) for 4 weeks	BSDS	General stimulant effects on sleep as recorded by BSDS (Mean ± SD): T1 = 22.58 ± 11.36; T2 = 20.29 ± 9.25; MPH (Mean ± SD): T1 = 21.79 ± 12.28 T2 = 19.00 ± 9.06 MOD (Mean ± SD): T1 = 25.60 ± 7.02 T2 = 25.20 ± 9.20 Nil significant improvements noted for sleep disturbance following intervention (p = 0.19)
Gehring et al. (2020) [34]	n = 32 (14 M, 18 F) Clinically stable glioma patients Exercise group (n = 21): Mean age = 49.2 ± 8.9 years; majority WHO II tumours (71%); mean tumour duration = 7.6 ± 5 years Control group (n = 11): Mean age = 48 ± 11.9 years; majority WHO II tumours (55%); mean tumour duration = 8.5 ± 8.6 years	Explore the possible impact of an exercise intervention designed to improve cognitive functioning in glioma patients and to assess cognitive test performance and PROs	6-month, individualised, home-based, aerobic exercise program: 3 × sessions conducted per week, based on baseline cardiorespiratory fitness and exercise tolerance testing Session duration 20–45 min at 60–85% of HR max, with duration and intensity progressed over the months Patients chose 1 of more activity (e.g., running, cycling, swimming) as long as they were able to meet their individual prescription	PSQI	Small benefit observed in sleep represented by + 0.34 (95% CI − 0.23–0.91) between-group difference following intervention
Hansen et al. (2020) [35]	n = 64 (44 M, 20 F) Functionally independent glioma patients; n = 32 included in intervention group, n = 32 in control group	Assess the effectiveness of physical and occupational therapy rehabilitation interventions compared with usual care for quality of life during treatment	Random assignment to supervised rehab vs usual care Intervention included 3 × sessions per week (M,W,F) immediately following participants radiation treatment 90 min intervention performed in a group setting Cardiovascular loads up to 75% oh HRR, resistance training of 3 continuous series of exercises with loads progressing from 70 to 75% of 1RM, progressing from 12 to 10 reps Intervention also included 15 min of individual physical therapy for specific deficits/impairments	EORTC QLQ-C30 & BN20	*Intention to treat group (n* = *64) analysis* Insomnia: β-coefficient = 10.8, p = 0.20 Drowsiness: β-coefficient = 12.3, p = 0.04 *Complete Case Population (n* = *55)* Insomnia: Intervention Mean = 17.3; Control Mean = 27.7, β-coefficient = 10.4, p = 0.21 Drowsiness: Intervention Mean = 29.2; Control Mean = 41.3, β-coefficient = − 12.1, p = 0.04
Milbury et al. (2018) [38]	n = 5 dyads (3 M, 7 F) HGG patients and their family caregivers Patients mean age = 51.94 ± 20.20 years, Caregivers mean age: 58.16 ± 10.15 years	Establish the feasibility of a dyadic yoga program for newly diagnosed HGG patients and their family caregivers targeting QOL outcomes	2–3 × 60 min guided yoga sessions over the course of the newly diagnosed HGG patients’ radiation therapy Vivekananda Yoga	PSQI	*Mean* ± *SD Pre-intervention PSQI Scores* PSQI patients = 10.75 ± 2.06 Caregivers = 10.40 ± 2.07 *Post-intervention* ↓Mean ± SD PSQI patients 8.00 ± 1.14, Cohens *d* = 1.17 ↑Mean ± SD PSQI Caregivers 11.20 ± 3.03, Cohens *d* = 0.49
Schloss et al. (2021) [37]	n = 83 (42 M, 41 F) HGG patients, mean age of 53.3 ± 12.6 years; majority males (50.6%) with GBM diagnosis (90%)	Investigate the use of cannabis in two different ratios and assess the effect on PROs	1:1 and 4:1 ratio of THC:CBD (1:1 THC 4.6 mg/ml:CBD 4.8 mg/ml and 4:1 THC 15 mg/ml:CBD 3.8 mg/ml) 12-week intervention in conjunction with standard with follow up appointments occurring at 4, 8 and 12 weeks	Fact-BR	*Mean* ± *SD Sleep scores* Baseline sleep = 2.3 ± 1.4 Week 4 = 3 ± 1.1 Week 8 = 3.3 ± 0.82 Week 12 = 3.3 ± 0.87 Overall improvement in sleep reported for both intervention groups (p = 0.012) Results in favour of the 1:1 ratio over the 4:1 ratio

*CSA* Central Sleep Apnea; *ESS* Epworth Sleepiness Scale; *GBM* Glioblastoma Multiforme; *BSDS* Brief Sleep Disturbance Scale; *HGG* High-Grade Glioma; *OSA* Obstructive Sleep Apnea; *PSG* Polysomnography; *PSQI* Pittsburgh Sleep Quality Index; *WHO* World Health Organisation

### Types, prevalence, and severity of sleep disturbance in PBT survivors

Sleep disturbance was described using a variety of sleep-related terms, mainly related to dissatisfaction with sleep quality, insomnia (disruption in sleep quantity, pattern, or architecture), somnolence, and obstructive sleep apnea; of these, insomnia and dissatisfaction with sleep quality were the highest reported sleep problems as most studies assessed these types of sleep complaints. Sleep-related questions in HRQoL studies asked specifically about disturbed sleep or insomnia. Across studies reporting validated sleep measures and validated symptom or outcome assessments (Table 1), few reported the prevalence of sleep disturbance assessed by clinical thresholds or cut-offs for respective questionnaires. Of five studies that used the PSQI, three reported the percentage of people scoring > 5 (i.e., significant sleep disturbance) with results estimating the prevalence of sleep disturbance between 37.0 and 81.8% [19, 21, 29]. One study using the GSDS [17] reported 100% of subjects experienced sleep disturbance. Two studies using the NCCN Distress Thermometer [20] and the CTCAE [26] reported the prevalence of sleep disturbance in their samples of people with glioma at 33.3% and 46.8%, respectively. Regarding HRQoL studies (Table 2), one study using the MDASI-BT reported the prevalence of sleep disturbance as 22% and drowsiness as 28% [14]. Additionally, mean PSQI scores reported in five studies ranged from 5.19 ± 3.39 to 11.53 ± 5.38, indicative of significant sleep disturbance [19, 21, 29, 33, 37]. Of two studies reporting the ISI, one reported that 17.4% of PBT survivors experienced moderate insomnia, while a further 4.1% experienced severe insomnia [29]. The single study utilising PSG reported that the average total sleep time of PBT survivors ranged between 309–319 min (5.15–5.50 h) per night [38].

### Risk factors/clinical associations

Treatment modalities such as surgical resection [19, 27, 39, 42], radiotherapy [30, 35, 38, 45], and corticosteroid use [29] appear to be associated with sleep disturbance in PBT survivors. Undergoing surgical resection was associated poorer sleep quality and sleep disturbance in short-term, post-operative follow-ups (e.g., 1–12 months) [39, 42] compared to longer term follow-up periods (e.g., > 12 months) [19, 27]. However, a longitudinal study following surgical resection for LGG reported statistically significant sleep impairments at post-operative intervals from 12 to 72 months and 120 + months (*p* =  < 0.05) [27]. While several studies suggest that radiotherapy is associated with sleep disturbance, one study conducted during radiotherapy treatment found no difference in objectively and subjectively measured sleep when comparing pre-treatment to post-treatment up to 2 months[38]. However, another found that patients who receive higher doses of radiotherapy experience higher levels of insomnia and fatigue post-radiotherapy (*p* = 0.05) [35]. Finally, consistent with previous literature, the results from the HRQoL studies highlighted the occurrence of sleep disturbance in a symptom cluster with other prevalent symptoms, such as fatigue, drowsiness, stress, and pain [17, 18, 27, 45].

Two studies explored whether tumour characteristics such as location or laterality impacted sleep regulation or QoL; however, neither location and/or laterality appeared to be associated with sleep disturbance in the current review [18, 21]. Only one study (n = 5) reported caregiver sleep [37], thus limiting the ability to explore risk factors or clinical associations for sleep disturbance in caregivers specifically.

### Interventions

No evidence of the development or utilisation of any interventions to manage sleep symptoms in people affected by PBT was found. Five intervention studies that reported sleep data were deemed suitable based on the eligibility criteria (Table 3). These interventions included pharmacological [32], alternative therapy (i.e., cannabis) [36], and physical activity [33, 34, 37].

An open-label pilot study aimed to assess the general and differential efficacy of methylphenidate and modafinil in PBT survivors [32]. Following randomisation to one of three conditions and four-weeks of intervention, no significant improvements were noted in sleep disturbance.

A RCT investigated the use of cannabis in two different ratios of THC to CBD [36]. Overall, there was an improvement in sleep reported for both intervention groups compared to baseline, though results favoured the 1:1 ratio compared to the 4:1 ratio (Fact-BR Sleep mean group difference = 2.59; 95%CI = 2.4–2.77; *p* = 0.009).

Gehring et al. [33] performed a RCT that explored the impact of an individualised, home-based aerobic exercise program on cognitive functioning in glioma patients. The results from this trial found a small between group difference in PSQI results (+ 0.34; 95%CI − 0.23–0.91) favouring the intervention group [33]. Similarly, Hansen et al. [34] performed a RCT assessing the effectiveness of physical and occupational therapy interventions compared to usual care on QoL in glioma patients during treatment that were functionally independent. The intervention group displayed better insomnia (Intervention Mean = 17.3; Control Mean = 27.7, β-coefficient = 10.4, *p* = 0.21) and drowsiness (Intervention Mean = 29.2; Control Mean = 41.3, β-coefficient = − 12.1, *p* = 0.04) scores as measured by the EORTC QLQ-C30 and BN20 questionnaires when compared to the control group [34]. Finally, Milbury et al. [37] performed a single arm pilot study that involved a 12-session dyadic yoga program during radiation therapy for HGG survivors. Their results indicated a clinically significant reduction in patient sleep disturbance, as recorded by the PSQI, at the end of the 12 sessions (Baseline = 10.75 ± 2.06; post-intervention = 8.00 ± 1.41*; p* = 0.10, *d* = 1.17), though the opposite effect was noted in caregivers (Baseline PSQI = 10.40 ± 3.03; post-intervention = 11.20 ± 3.03; *p* = 0.34, *d* = 0.49) [37].

## Discussion

Sleep is an essential biological process with a vital role in maintaining homeostatic mechanisms, physiological function and psychological wellbeing [49–51]. It is critical to maximising health and QoL in people affected by cancer (i.e., cancer survivors and caregivers) [50, 51]. However, disturbances and alterations in sleep are commonly reported in people affected by cancer, with sleep disturbance observed in 50–60% of cancer survivors [1, 52] and 40–70% of caregivers [53]. Recently, two reviews highlighted sleep disturbance as a highly prevalent and severe symptom experienced by people diagnosed with brain tumours, significantly impacting HRQoL [6, 7]. Our systematic review extends on these findings by assimilating the current evidence regarding sleep disturbance in PBT survivors, and the first systematic review to explicitly explore sleep disturbance in adults affected by PBT (i.e., PBT survivors and their caregivers). Most studies (29 of 34) included in this systematic review had a primary focus on HRQoL, particularly frequently reported symptoms, levels of physical/psychological functioning, and overall QoL.

Our review demonstrates that sleep disturbance is highly prevalent and reported as one of the most common symptoms experienced by PBT survivors. While it is difficult to determine the prevalence of sleep disturbance from results of the other HRQoL studies, sleep disturbance and drowsiness were repeatedly reported among the most prevalent and severe symptoms. The severity of sleep disturbance and drowsiness scored similarly despite the heterogeneity of PBT survivors participating in the included studies, as represented by the mean scores for the EORTC QLQ-C30 & BN20 and the MDASI-BT (Table 2). Consistent with previous literature, treatment modalities such as surgical resection [19, 27, 39, 42], radiation therapy [30, 35, 38, 45], and corticosteroid use [29] are associated with sleep disturbance in PBT survivors. Furthermore, sleep disturbance often appeared in a “symptom cluster” (i.e., consisting of two or more symptoms related to each other) with other prevalent symptoms such as fatigue, drowsiness, distress, and pain [17, 18, 29, 45]. Sleep disturbance is multi-faceted; psychologically and biologically driven sleep disturbance is bi-directional, each contributing to the perpetuation and exacerbation of sleep disturbance. Spielman’s 3P model of insomnia, also referred to as the behavioural model of insomnia, offers an illustrative paradigm that conceptualizes the multi-faceted phenomenon of sleep disturbance [54]. The model suggests that predisposing (e.g., bio-psychological factors such as the presence of hyper-arousability, worry, prior personal/family history of sleep disturbance) and precipitating (e.g., acute occurrences such as hospitalisation, surgery, chemotherapy, radiation, corticosteroids) factors result in the development of insomnia, while perpetuating factors (e.g., actions that arise as a result of insomnia/sleep disturbance, such as maladaptive sleep behaviours, faulty beliefs/perceptions) result in insomnia becoming chronic [54]. As such, sleep disturbance may arise as a result *of* other common symptoms (e.g., pain, fatigue) or result *in* increased susceptibility to these other symptoms. However, there are a range of pathophysiological processes underpinning sleep disturbance in PBT survivors (e.g., direct tumour and treatment influences, unique susceptibility to heterogenous symptoms, cognitive impairment, neuropsychological deficits), and further research is required to truly understand the nature of sleep disturbance in this population.

The caregiver-survivor dyad is a critical resource in ongoing care of PBT survivors. Providing care for people diagnosed with PBTs may present an increased burden relative to other cancers due to the complexity of presentation throughout disease trajectory. The caregiver’s role of support and management of the person with PBT is unique as they must manage cognitive deterioration, personality change, disinhibition, and communication difficulties, while sustaining a meaningful relationship with the person who has usually been a major part of their lives for a significant period of time [6, 55]. Despite the importance of this role, there is a distinct lack of inclusion of caregivers in PBT research, with our review identifying only one study involving caregivers [37].

While there is a plethora of sleep-focused literature reporting a myriad of interventions to combat sleep disturbance in other populations, our review could not identify evidence for any such interventions in people with PBT and their caregivers. CBT is defined as interventions/approaches that help individuals identify helpful and/or maladaptive thoughts, feelings, and perceptions and implement beneficial coping behaviours [56]. Despite being recommended as a front-line treatment for sleep disturbance in cancer populations in general [56], CBT was not studied as an intervention in any of the included manuscripts. Notwithstanding the absence of these sleep-focused interventions, our review found preliminary evidence suggesting that physical activity may elicit beneficial outcomes on sleep disturbance, drowsiness, and fatigue specific to PBT survivors [22, 33, 34, 37]. The physical implications of a PBT diagnosis compromise QoL and independence, with marked reductions in strength and fitness when compared to age- and sex-matched normative data [57, 58]. Following a PBT diagnosis, reduced physical activity levels are observed, potentially exacerbating tumour- and treatment-related effects [57, 59]. However, higher levels of physical activity post-diagnosis may be associated with improved health outcomes, particularly outcomes that influence QoL, potentially leading to improved management of sleep disturbance and fatigue [57, 59]. In addition to physical activity, use of medicinal cannabis may also be an applicable intervention to assist with managing QoL concerns, particularly sleep disturbance in PBT survivors [36]. While the safety and tolerability of cannabis has been trialled in the general population, further research is required in the context of PBT before recommendations can be made.

The current systematic review is not without limitations. Studies recruited heterogenous samples within and between studies reporting a range of clinical and treatment features. This made it hard to identify prevalent types or presentations of sleep disturbance, as well as clear associations with other factors. Furthermore, most evidence stems from HRQoL research as secondary, single-item symptom scales. Small sample sizes in these studies resulted in a lack of statistical power. Studies in this population are subject to high attrition rates, particularly for people with HGG. As such, this results in a selection bias towards “more well” individuals typically remaining in study populations, meaning that overall performance of the population with regard to HRQoL domains may be over-estimated.

## Implications for practice and research

Understanding sleep disturbance in PBT survivors and their caregivers is important for providing quality care, yet current knowledge is limited. While there is a growing body of evidence linking sleep with overall health, sleep disturbance does not typically occur in isolation in PBT survivors and their caregivers, making prioritisation and management difficult. More research utilising standardized, multifaceted sleep assessment tools are needed to establish a better understanding of the patterns and prevalence of sleep disturbance. Most available evidence is limited to describing the presence of sleep disturbance subjectively as part of overall HRQoL evaluation; the use of objective assessments, such as PSG or actigraphy is imperative to establishing and understanding the patterns and manifestations of sleep disturbance in the context of PBT. However, the value of validated and brief sleep-focused questionnaires (e.g., PSQI, ISI) should not be overlooked as practical additions to improve longitudinal assessment and monitoring of symptoms over the disease trajectory. Finally, a plethora of sleep-focused research employ interventions such as CBT, mindfulness, and pharmacological interventions, however there is no evidence available in the context of PBT. Our systematic review does, however, provide preliminary evidence to suggest that physical activity may be beneficial to PBT survivors not only in the context of HRQoL, but for sleep specifically. Further research exploring other interventions with a focus on sleep disturbance in people affected by PBT is warranted including CBT.

## Supplementary Information

Below is the link to the electronic supplementary material.

Supplementary file1 (XLSX 39 KB)

Supplementary file2 (DOCX 16 KB)

## Data Availability

All data generated or analysed during this study are included in this published article and its supplementary information files.
