# Supplementary material for: Prevalence and management of sleep disturbance in adults with primary brain tumours and their caregivers: a systematic review
Source: J Neurooncol. 2023 Mar 2;162(1):25–44. doi: 10.1007/s11060-023-04270-1 (PMC10049936; doi:10.1007/s11060-023-04270-1)
Supplement: Supplementary file 2 — Supplementary file2 (DOCX 16 KB) [file 11060_2023_4270_MOESM2_ESM.docx]

| Pubmed | ("brain neoplasms"[MeSH Terms] OR brain cancer[Text Word] OR "Brain tumo*" OR "brain cancer*" OR "brain Metastasis" OR "brain Metastases" OR "brain neoplasm*" OR "cerebral cancer*" OR "cerebral tumo*" OR "Glioblastoma multiforme" OR Glioma* OR Neuroma* OR Meningioma OR Astrocytoma OR "Pituitary adenoma" OR Oligodendroglioma OR "Pineal region tumo*" OR Ependymoma OR Pineocytoma OR Pineoblastoma OR ((Brain OR Cranial) AND (tumo* OR Neoplasm OR Cancer))) AND (Caregiver OR carer OR family OR relative) AND ("sleep wake disorders"[MeSH Terms] OR Sleep OR "sleep quality" OR "sleep difficult*" OR "sleep disturbance*" OR "sleep pattern*" OR "sleep disorder*" OR "sleep deprivation" OR "sleep pattern disturbance*" OR "sleep apnoea" OR "sleep movement disorder*" OR (("Patient-reported outcome*" OR self-report* OR diaries OR diary) AND sleep) OR circadian OR sleep-rest OR sleep-wake OR parasomnia OR hypersomnia OR somnolence OR sleepiness OR insomnia OR polysomnography OR Actigraphy OR Actiwatch OR "sleep EEG" OR "REM sleep behavior disorder") |
| --- | --- |
| MEDLINE SEARCH | (MH "Brain Neoplasms+") OR (Brain tumo* OR brain cancer* OR brain Metastas*OR brain neoplasm* OR malignant brain tumo* OR cerebral cancer* OR cerebral tumo* OR Glioblastoma multiforme OR Glioma* OR ((Brain OR Cranial) AND (tumo* OR Neoplasm OR Cancer)) AND (Caregiver OR carer OR family OR relative)) AND (MH "Sleep Wake Disorders+") OR ( Sleep OR sleep quality OR sleep difficult* OR sleep disturbance* OR sleep pattern* OR sleep disorder* OR sleep deprivation OR sleep pattern disturbance* OR sleep apnoea OR sleep movement disorder* OR fatigue OR circadian OR sleep-rest OR sleep-wake OR parasomnia OR hypersomnia OR somnolence OR sleepiness OR insomnia OR polysomnography OR Actigraphy OR Actiwatch OR sleep EEG OR REM sleep behavio* disorder OR Oura Ring OR Fitbit OR Garmin ) NOT (animals [mh] NOT humans [mh]) |
| EMBASE SEARCH | ('sleep disorder'/exp OR sleep OR 'sleep quality' OR 'sleep difficult*' OR 'sleep disturbance*' OR 'sleep pattern*' OR 'sleep disorder*' OR 'sleep deprivation' OR 'sleep pattern disturbance*' OR 'sleep apnoea' OR 'sleep movement disorder*' OR (('patient-reported outcome*' OR 'self report*' OR diaries OR diary) AND sleep) OR circadian OR 'sleep rest' OR 'sleep wake' OR parasomnia OR hypersomnia OR somnolence OR sleepiness OR insomnia OR polysomnography OR actigraphy OR actiwatch OR 'sleep eeg' OR 'rem sleep behavior disorder') AND ('brain cancer'/exp OR 'brain tumo*' OR 'brain cancer*' OR 'brain metastasis' OR 'brain metastases' OR 'brain neoplasm*' OR 'cerebral cancer*' OR 'cerebral tumo*' OR 'glioblastoma multiforme' OR glioma* OR neuroma* OR meningioma OR astrocytoma OR 'pituitary adenoma' OR oligodendroglioma OR 'pineal region tumo*' OR ependymoma OR pineocytoma OR pineoblastoma OR ((brain OR cranial) AND (tumo* OR neoplasm OR cancer))) AND (caregiver OR carer OR family OR relative) |
| PsycInfo | DE "Brain Neoplasms" OR ( "Brain tumo*" OR "brain cancer*" OR "brain Metastasis" OR "brain Metastases" OR "brain neoplasm*" OR "cerebral cancer*" OR "cerebral tumo*" OR "Glioblastoma multiforme" OR Glioma* OR Neuroma* OR Meningioma OR Astrocytoma OR "Pituitary adenoma" OR Oligodendroglioma OR "Pineal region tumo*" OR Ependymoma OR Pineocytoma OR Pineoblastoma OR ((Brain OR Cranial) AND (tumo* OR Neoplasm OR Cancer)) AND (Caregiver OR carer OR family OR relative) ) AND DE "Sleep Wake Disorders" OR Sleep OR "sleep quality" OR "sleep difficult*" OR "sleep disturbance*" OR "sleep pattern*" OR "sleep disorder*" OR "sleep deprivation" OR "sleep pattern disturbance*" OR "sleep apnoea" OR "sleep movement disorder*" OR (("Patient-reported outcome*" OR self-report* OR diaries OR diary) AND sleep) OR circadian OR sleep-rest OR sleep-wake OR parasomnia OR hypersomnia OR somnolence OR sleepiness OR insomnia OR polysomnography OR Actigraphy OR Actiwatch OR "sleep EEG" OR "REM sleep behavior disorder" |
| CINAHL | (MH "Brain Neoplasms+") OR "Brain tumo*" OR "brain cancer*" OR "brain Metastasis" OR "brain Metastases" OR "brain neoplasm*" OR "cerebral cancer*" OR "cerebral tumo*" OR "Glioblastoma multiforme" OR Glioma* OR Neuroma* OR Meningioma OR Astrocytoma OR "Pituitary adenoma" OR Oligodendroglioma OR "Pineal region tumo*" OR Ependymoma OR Pineocytoma OR Pineoblastoma OR ((Brain OR Cranial) AND (tumo* OR Neoplasm OR Cancer)) AND (Caregiver OR carer OR family OR relative) AND (MH "Sleep Disorders+") OR Sleep OR "sleep quality" OR "sleep difficult*" OR "sleep disturbance*" OR "sleep pattern*" OR "sleep disorder*" OR "sleep deprivation" OR "sleep pattern disturbance*" OR "sleep apnoea" OR "sleep movement disorder*" OR (("Patient-reported outcome*" OR self-report* OR diaries OR diary) AND sleep) OR circadian OR sleep-rest OR sleep-wake OR parasomnia OR hypersomnia OR somnolence OR sleepiness OR insomnia OR polysomnography OR Actigraphy OR Actiwatch OR "sleep EEG" OR "REM sleep behavior disorder" |
| PsychINFO | Sleep OR "sleep quality" OR "sleep difficult*" OR "sleep disturbance*" OR "sleep pattern*" OR "sleep disorder*" OR "sleep deprivation" OR "sleep pattern disturbance*" OR "sleep apnoea" OR "sleep movement disorder*" OR (("Patient-reported outcome*" OR self-report* OR diaries OR diary) AND sleep) OR circadian OR sleep-rest OR sleep-wake OR parasomnia OR hypersomnia OR somnolence OR sleepiness OR insomnia OR polysomnography OR Actigraphy OR Actiwatch OR "sleep EEG" OR "REM sleep behavior disorder" AND "Brain tumo*" OR "brain cancer*" OR "brain Metastasis" OR "brain Metastases" OR "brain neoplasm*" OR "cerebral cancer*" OR "cerebral tumo*" OR "Glioblastoma multiforme" OR Glioma* OR Neuroma* OR Meningioma OR Astrocytoma OR "Pituitary adenoma" OR Oligodendroglioma OR "Pineal region tumo*" OR Ependymoma OR Pineocytoma OR Pineoblastoma OR ((Brain OR Cranial) AND (tumo* OR Neoplasm OR Cancer)) AND (Caregiver OR carer OR family OR relative) |
